# The feasibility analysis of calculating proptosis by simple Heron’s formula

**DOI:** 10.1371/journal.pone.0234016

**Published:** 2020-06-01

**Authors:** Weili Zhang, Qinying Huang, Yile Chen, Jinying Li

**Affiliations:** Department of Ophthalmology, Peking University Shenzhen Hospital, Shenzhen, Guangdong, China; Faculty of Medicine, Cairo University, EGYPT

## Abstract

**Purpose:**

We propose a new method to calculate proptosis by using the simple Heron’s formula and analyze its feasibility.

**Method:**

It was a none-inferiority trial. The registration number was ChiCTR1900026490. The absolute value of proptosis in 120 eyes, 60 patients without eye injury or diseases, was measured by computed tomography (CT) and simple Heron’s formula. We did regression analysis and analyzed the differences between the two methods with Medcalc software version 19.0.4. The result was showed by Passing–Bablok regression analysis diagram and Bland and Altman plot.

**Results:**

The Passing-Bablok showed that the result of proptosis measured by CT and simple Heron’s formula showed good positive correlation. A 95% limit of agreement in proptosis between CT and Heron’s formula method was -0.46 to 0.54 mm in right eye and -0.45 to 0.46 mm in left eye. 1.66% (1/60) point was outside 95% LoA in both eyes. Moreover, a 95% limit of agreement between CT and Heron’s formula method was -0.42 to 0.56 mm in difference of both eyes. 3.33% (2/60) points were outside 95% LoA. The points in all Bland and Altman plots were lower than 5%. It means that the results of comparison between the two methods had a good consistency in the measurement of proptosis.

**Conclusions:**

Heron’s formula could be applied to calculate proptosis and has a good consistency compared with computed tomography (CT). This method is practical in proptosis assessment because of its accuracy, reliability and simplicity.

## 1. Introduction

Proptosis is the vertical distance from anterior aspect of the lateral orbital margin to the apex of the cornea. It can be classified into three types, including relative proptosis (comparison between the right eye and left eye), comparative proptosis (compared with earlier measurements in the same eye) and absolute proptosis (compared with ‘‘normal” values in the general population) [1]. Clinically, the value of proptosis plays an important role in the diagnosis, treatment and follow-up of orbital diseases. Exophthalmometry is a method to quantify the degree of proptosis. It is influenced by the discrepancy of orbital bony volume and its content. There are various kinds of exophthalmometers using for proptosis measurement, but the results are different because of the different clinical experiences of doctors [2–4]. At present, Hertel exophthalmometer is a common method to measure proptosis, but its credibility and accuracy are not satisfied [5]. It is suggested that the orbital computed tomography (CT) is the gold standard to measure proptosis [6]. However, it cannot be used widely in clinical work because it has high radiation and high price. Meanwhile, the approach of measuring proptosis by Heron’s formula has not been reported yet. In our study, we explored the value of Heron’s formula in clinical application. And we compared its accuracy with CT measurement.

## 2. Methods

### 2.1 Objects

It was a none-inferiority trial. 120 eyes in 60 Asian patients without orbital and maxillofacial diseases were selected. They came to the Ophthalmology Department of Peking University Shenzhen Hospital from January 2019 to December 2019. The participants, including 34 males and 26 females, were aged from 24 to 63 years old, with a mean age of 44.8 years old. Exclusion criteria were binocular high myopia, strabismus, head and face fractures and developmental abnormalities. For all patients, orbital CT and Heron’s formula were used to calculate the absolute proptosis value of left and right eye respectively, and the relative difference between two eyes was also calculated. Authors had access to get information of patients that could identify individual participant during or after data collection. The individual in this manuscript has given written informed consent (as outlined in PLOS consent form) to publish these case details. The study was approved by the committee of Peking University Shenzhen Hospital in accordance with the Helsinki declaration.

### 2.2 Experimental methods and technical control

#### 2.2.1 Orbital CT was used to quantify the absolute value of proptosis of both eyes and then we calculated the relative difference value

**Fig 1:**
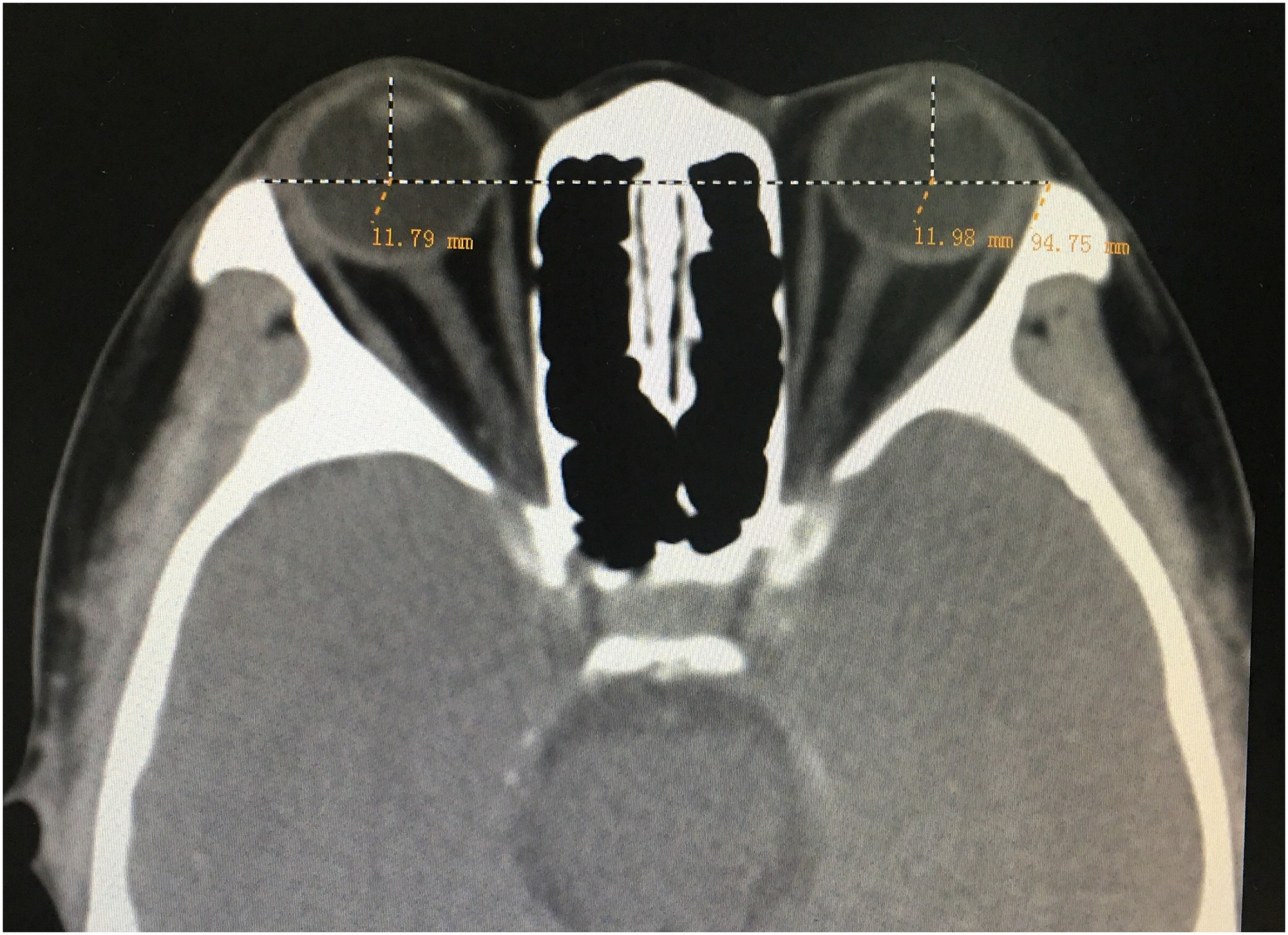
It was the transverse section of head scanning by computed tomography. An interzygomatic line was drawn on the axial view image when the lens was seen clearly. A perpendicular line was drawn from the inner corneal surface to the interzygomatic line, bisecting the lens.

The subjects were in supine position with eyes closed, and the orbit was scanned at 1mm thickness continuously with CT (SOMATOM PIUS 4 POWER) by the same operator. When the center of the lens and the whole optic nerve were showed synchronously in the orbital axis image, we selected it as the standard measurement plane [7]. The connection of the lateral orbital margins in a horizontal plane was drawn under the soft tissue window and a perpendicular line was drawn from the inner corneal surface. The length of this perpendicular line was taken as the proptosis (Fig 1). The measurements were taken three times by the same observer. Finally, the mean value of proptosis and the relative difference between both eyes were calculated.

#### 2.2.2 We supposed three points of the triangle that was made with one of corneal apex and bilateral orbital margin

**Fig 2:**
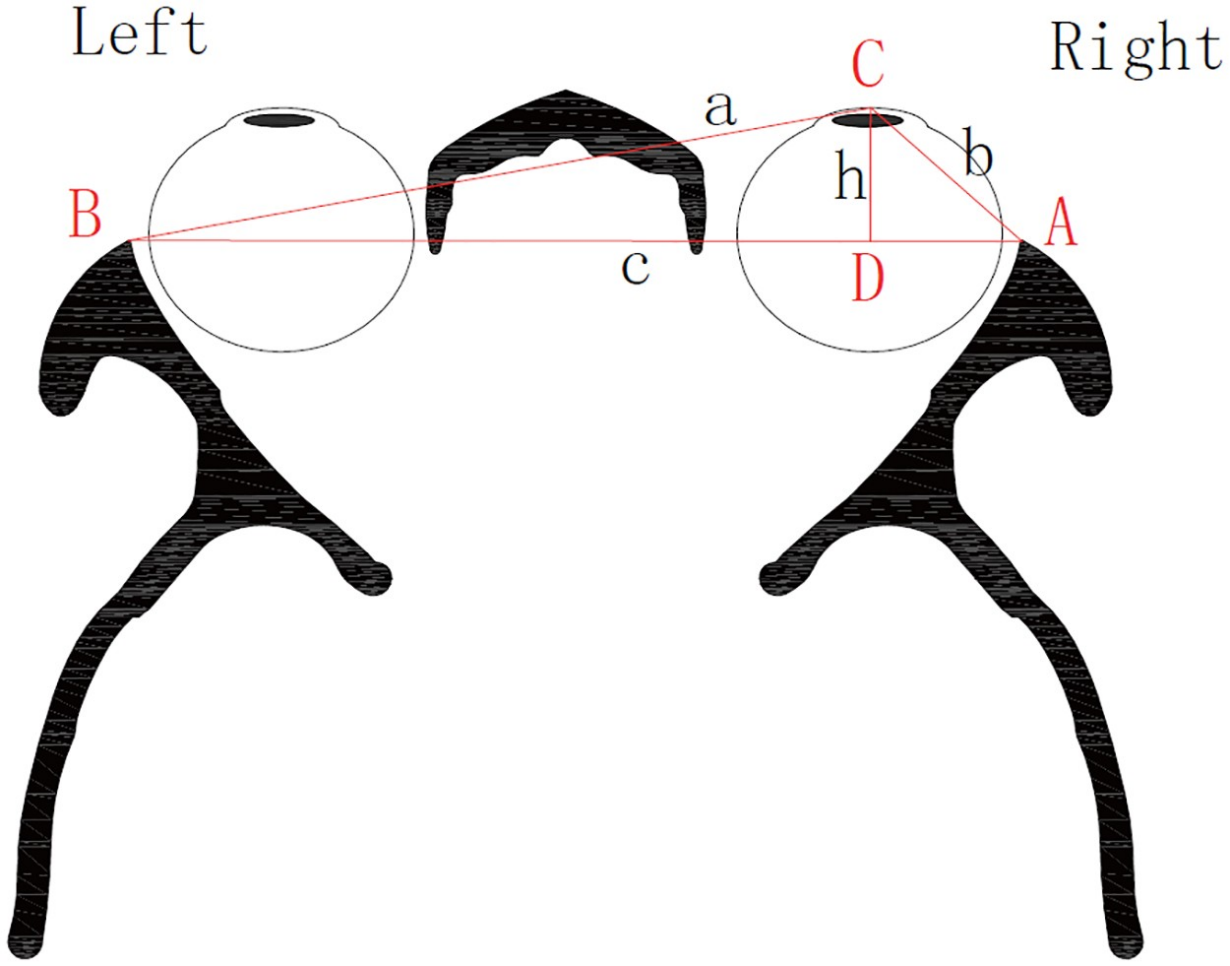
The simplified image of the transvers section of head scanning by computed tomography. Taking the corneal apex as point C, bilateral margin as points A and B. Point D is a crossing point between vertical line from point C and line AB. Define AB = c, AC = b, BC = a, and h = CD.

The distances from corneal apex to lateral orbital margin were considered as ‘a’ and ‘b’ respectively. And the length between bilateral orbital margin was regarded as ‘c’. ‘p’ was the half value of perimeter. According to the Heron’s formula, the area of triangle is S = √[p(p-a)(p-b)(p-c)]. What’s more, the area S can also be calculated by the following formula: S = ch/2 (‘h’ was the height of triangle when ‘c’ was considered as the base line). We concluded that √[p(p-a)(p-b)(p-c)] = ch/2. Obviously, the proptosis can be expressed by the value of ‘h’ (Fig 2).

**Fig 3:**
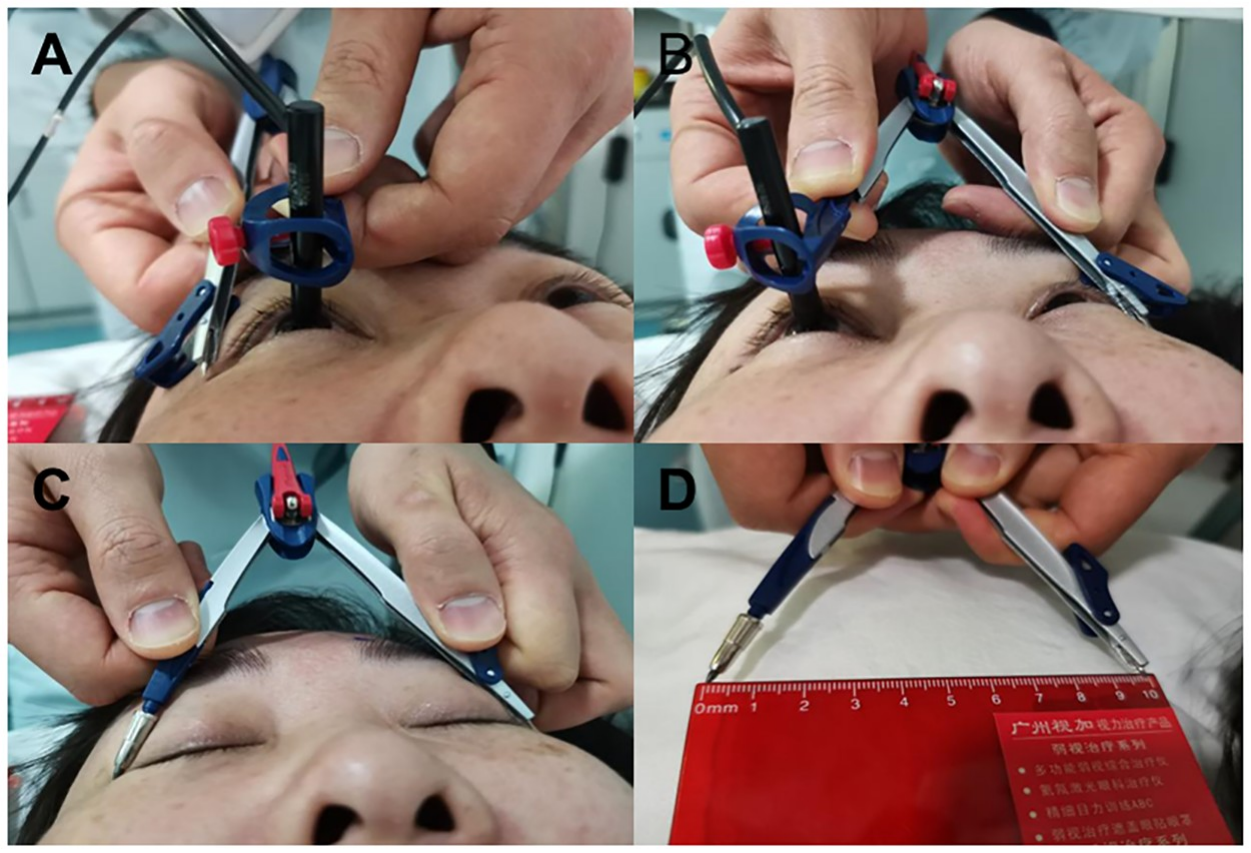
Using the Amplitude-mode Ultrasound probe and compasses to measure the distance from corneal apex to bilateral orbital margin (A and B). Measuring the transverse distance between the bilateral orbital margin marker (C). The distance was measured with a ruler (D).

Heron’s formula was used to calculate the absolute value of proprtosis in both eyes and the relative difference value between two eyes was calculated, too. The subjects were in supine position and the lowest points of bilateral orbital outer edge were marked with marker pen. Procaine hydrochloride eye drops (Alcon registration, Registered number H20160133) were applied to both eyes every five minutes for three times in total. 70% alcohol was used to clean the Amplitude-mode Ultrasound probe and the probe was fixed with one foot of compasses. We used the infra-red Amplitude-mode Ultrasound probe to guide the subjects looking straight ahead and the probe was touched the corneal central apex lightly. Another foot of the compasses was placed on the homolateral side marker point of the orbital lateral margin (Fig 3A). The distance was regarded as AC = b (mm). Similarly, when the infra-red Amplitude-mode Ultrasound probe was on the corneal apex, another foot of the compasses was placed on the heterolateral side marker point of the orbital lateral margin (Fig 3B). The distance was regarded as BC = a (mm). Then two feet of the compasses were placed on bilateral marker points of the orbital margin (Fig 3C). The distance was regarded as AB = c (mm). We used a ruler to measure the above distance and the all length of triangle was showed out. Later, we set up the formula in Excel and input the data to calculate the value of proptosis. Easily, both absolute value and the relative difference value of proptosis of eyes were calculated. The data was recorded as the Heron’s formula methodology. In order to minimize the measurement error, the measurements were taken three times by the same observer and the mean value of proptosis was calculated.

### 2.3 Statistical methods

SPSS 19.0 statistical software was used to conduct homogeneity test of variance and normal distribution. The absolute value and relative difference of binoculus proptosis obtained by CT and Heron’s formula were satisfied. Then, Medcalc software version 19.0.4 was used to conduct Passing-Bablok regression analysis and Bland and Altman plot with data obtained by the above methods.

## 3. Results

**Fig 4:**
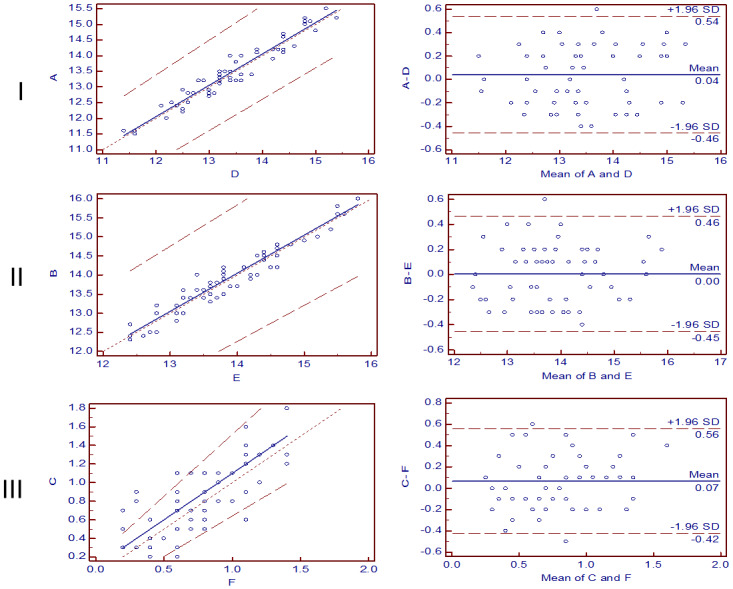
(I) Method comparison of A and D, presented by Passing-Bablok regression analysis (column 1) and Bland and Altman plot, given the confidence interval of 95% (column 2). A and D represent the values of proptosis of right eye measured by CT and Heron’s formula respectively. (II) Method comparison of B and E, presented aby Passing-Bablok regression analysis (column 1) and Bland and Altman plot, given the confidence interval of 95% (column 2). B and E represent the values of proptosis of left eye measured by CT and Heron’s formula respectively. (III) Method comparison of C and F, presented by Passing-Bablok regression analysis (column 1) and Bland and Altman plot, given the confidence interval of 95% (column 2). C and F represent difference of proptosis between two eyes measured by CT and Heron’s Formula respectively.

The absolute proptosis of right eye measured by CT and Heron’s formula was 13.44±0.96mm and 13.4±0.94mm respectively, while the left eye was 13.92±0.84mm and 13.91±0.83mm. Besides, the differences between two eyes were 0.80±0.36mm and 0.73 ±0.33mm. The Passing-Bablok regression analysis showed that the regression equation of proptosis of right eye measured by CT and Heron’s formula method is y = 0.05 + 1.00 x. The intercept A is 0.05 (95% CI: -1.39 to 0.05) and the slope B is 1.00 (95% CI: 1.00 to 1.11). A 95% limit of agreement between CT and Heron’s formula method was -0.46 to 0.54 mm in right eye proptosis (Fig 4I). The Passing-Bablok regression analysis showed that the regression equation of proptosis of left eye measured by CT and Heron’s formula method is y = 0.05 + 1.00 x. The intercept A is 0.05 (95% CI: -1.04 to 0.76) and the slope B is 1.00 (95% CI: 0.95 to 1.08). A 95% limit of agreement between CT and Heron’s formula method was -0.45 to 0.46 mm in left eye proptosis (Fig 4II). The Passing-Bablok regression analysis showed that the regression equation of difference proptosis between two eyes measured by CT and Heron’s formula method is y = 0.10 + 1.00 x. The intercept A is 0.05 (95% CI: -0.23 to 0.19) and the slope B is 1.00 (95% CI: 0.88 to 1.33). 1.66% (1/60) point was outside 95% LoA in both eyes. Moreover, a 95% limit of agreement between CT and Heron’s formula method was -0.42 to 0.56 mm in difference of both eyes. 3.33% (2/60) points were outside 95% LoA (Fig 4III). The points in all Bland and Altman plots were lower than 5%, which means the results of comparison between these two methods had a good consistency in the measurement of proptosis.

## 4. Discussions

The results of Passing-Bablok regression analysis showed that the proptosis of two eyes or the difference proptosis between two eyes measured by CT and Heron’s formula had good correlation. And the result of Bland and Altman plots analysis showed no significant difference in the measurement of the absolute value and relative difference value of two eyes between CT and Heron’s formula in normal people. It suggested that the accuracies of two methods are comparative in measuring proptosis without any abnormity. Delmas et al. has demonstrated that CT has the same accuracy with Hertel exophthalmometer [8], but the Heron’s formula has not been reported yet.

Proptosis is defined as protrusion of the eyeball anteriorly out of the orbit. Of all the methods that have been proposed to evaluate globe position, the Hertel exophthalmometer is the most widely used equipment [5, 9]. However, previous studies have found limitations in the reproducibility of its measurements, particularly among different observers [2, 9–12]. The differences in measurement that arise with this device may result from asymmetry of the lateral orbital rims, compression of soft tissues, parallax errors and the lack of a uniform measuring technique. In contrast, CT of the orbits provides more accurate values compared with exophthalmometry. In an investigation to explore the range of proptosis values in the Korean population, Kim et al. emphasized the use of CT to achieve a more precise evaluation [6]. Compared with Two-Dimensional (2D) CT, Three-Dimensional (3D) CT is more accurate in identifying the corneal apex and it can correct the corneal apex deviation in patients with vertical strabismus. 3D CT also provides important information for surgical design [13–16]. CT is the recommended standard examination and diagnostic technique for orbital lesions, especially for patients with orbital fractures before surgery. However, because of the radioactivity, CT examinations are only used in cases requiring simultaneous understanding of the intraorbital structure [6]. The eyelid is difficult to distinguish from the anterior surface of the cornea in CT, while the posterior surface of the cornea can be clearly identified. Thus, there is about 0.5mm measurement error of corneal thickness when measuring from the posterior surface of the cornea [17]. Eyelids should be closed to avoid Bell’s phenomenon and measurement error. Because of the radiation damage and high price, CT is not accepted widely. Besides, the examination results of CT need to be analyzed further by software and it cannot be measured quickly, which also limits its application in clinical practice.

**Fig 5:**
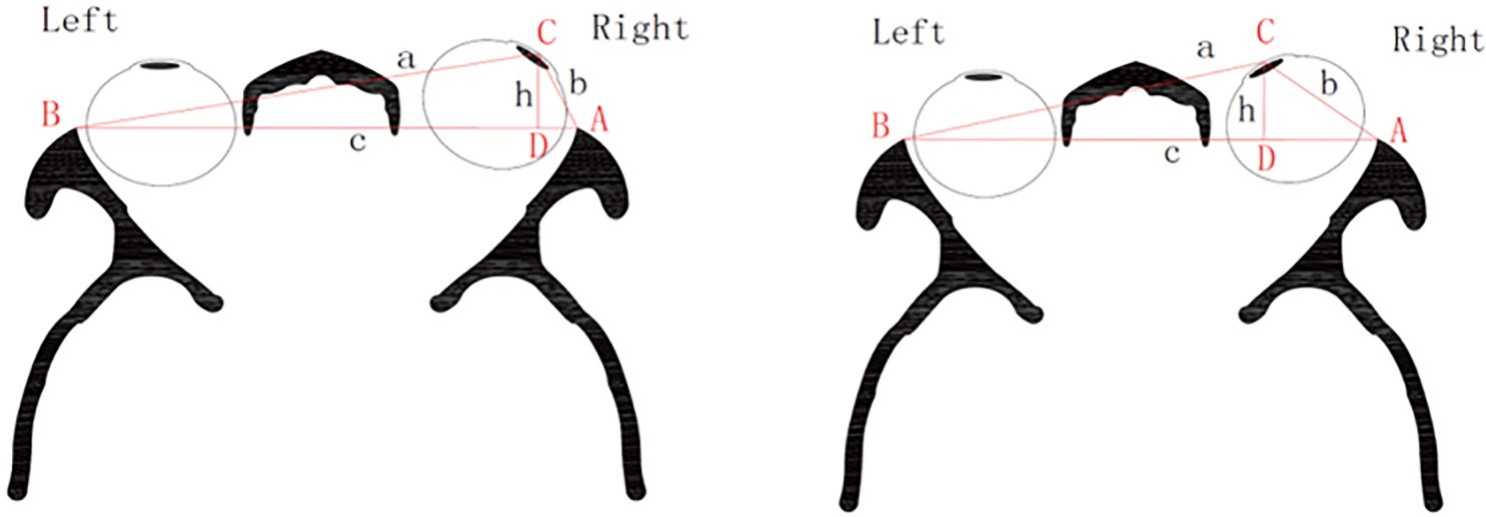
Measuring the horizontal non-axial proptosis based on Heron’s formula.

The measurement of proptosis by Heron’s formula has not been reported yet. Our study showed its accuracy was comparable to CT measurement in normal people. The advantages of this method were as follows: 1. The compasses foot, touching the corneal apex, was fixed with infra-red Amplitude-mode Ultrasound probe. The other compasses foot was placed on the lowest point of the lateral orbital margin. Point-to-point connection was used to measure the distance of bilateral orbital margin and the distance between the apex of the cornea and the lowest point of lateral orbital margin; 2. The operation of Heron’s formula was simple and convenient for preoperative, intraoperative and postoperative measurement. In the process of correction of proptosis and extraction of orbital tumors, the degree of proptosis can be accurately evaluated to guide the intraoperative surgical correction. 3. Applying the measurement criteria, we suggested that it could be used for measuring horizontal non-axial proptosis (Fig 5) and the degree of sunken eyeball could also be calculated with the Heron’s formula while the Hertel exophthalmometer cannot. 4. This technique is at low-cost and not time-consuming. It can available everywhere. 5. With the development of infrared measurement software, proptosis measured by touching equipment can be replaced gradually. The non-invasive, cheap and fast method will be applied by using mobile APP.

However, there were some disadvantages of this method. It required topical anesthesia and contacting with cornea when operating. Careful operation and strict disinfection of the probe were strict to avoid damage to the cornea. Compared with the method of Hertel exophthalmometer and CT, we also needed to take bilateral orbital margin as reference point for measurement. Measurement error did exist in people with unilateral orbital trauma and dysplasia. Imperfectly, we collected a small number of cases in this study and the subjects with orbital lesions were not included.

## 5. Conclusions

Heron’s formula can be applied to calculate proptosis by measuring the distance between different points and has a good consistency compared with computed tomography (CT). This method is practical in clinical use of proptosis assessment because of its accuracy, reliability and simplicity.

## 6. Disclosure

Ethics approval and consent to participate: The study was approved by the committee of Peking University Shenzhen Hospital in accordance with the Helsinki declaration.

Consent for publication: The individual in this manuscript has given written informed consent (as outlined in PLOS consent form) to publish these case details.

## Supporting information

S1 Data (DOCX)
