# Supplementary material for: The feasibility analysis of calculating proptosis by simple Heron’s formula
Source: PLoS One. 2020 Jun 1;15(6):e0234016. doi: 10.1371/journal.pone.0234016 (PMC7263846; doi:10.1371/journal.pone.0234016)
Supplement: S1 Data — (DOCX) [file pone.0234016.s001.docx]

| ID | CT (Proptosis) | | Heron’s formula (Proptosis) | |
| --- | --- | --- | --- | --- |
|  | Right eye | Left eye | Right eye | Left eye |
| 1 | 13.4 | 14.1 | 13.6 | 13.8 |
| 2 | 12.9 | 13.4 | 12.5 | 13.2 |
| 3 | 13.2 | 14 | 12.8 | 13.4 |
| 4 | 15.1 | 14.2 | 14.8 | 14.5 |
| 5 | 13.2 | 13.9 | 13.5 | 14.2 |
| 6 | 14.0 | 13.5 | 13.6 | 13.8 |
| 7 | 13.4 | 14.5 | 13.8 | 14.4 |
| 8 | 14.1 | 13.3 | 13.9 | 13.6 |
| 9 | 12.8 | 13.4 | 12.7 | 13.5 |
| 10 | 14.2 | 14.6 | 13.9 | 14.4 |
| 11 | 13.3 | 14.5 | 13.2 | 14.3 |
| 12 | 12.7 | 13.8 | 13.0 | 13.7 |
| 13 | 13.2 | 12.4 | 13.4 | 12.4 |
| 14 | 11.5 | 12.3 | 11.6 | 12.4 |
| 15 | 12.4 | 13.0 | 12.1 | 12.8 |
| 16 | 13.5 | 14.7 | 13.2 | 14.6 |
| 17 | 14.8 | 14.2 | 15.0 | 14.6 |
| 18 | 15.0 | 14.5 | 14.8 | 14.6 |
| 19 | 14.1 | 14.9 | 14.4 | 15.0 |
| 20 | 13.2 | 12.4 | 12.9 | 12.6 |
| 21 | 15.2 | 14.2 | 15.4 | 14.5 |
| 22 | 12.5 | 13.0 | 12.3 | 13.1 |
| 23 | 13.1 | 14.0 | 13.2 | 13.8 |
| 24 | 12.2 | 12.5 | 12.5 | 12.7 |
| 25 | 11.6 | 13.2 | 11.6 | 12.8 |
| 26 | 13.8 | 13.5 | 13.5 | 13.8 |
| 27 | 13.4 | 14.0 | 13.2 | 13.8 |
| 28 | 12.0 | 12.8 | 12.2 | 13.1 |
| 29 | 14.0 | 13.7 | 13.4 | 14.0 |
| 30 | 13.2 | 13.0 | 13.6 | 13.2 |
| 31 | 13.5 | 14.8 | 13.2 | 14.6 |
| 32 | 15.1 | 14.2 | 14.9 | 14.1 |
| 33 | 12.5 | 13.6 | 12.3 | 13.2 |
| 34 | 14.2 | 13.6 | 14.3 | 13.5 |
| 35 | 12.9 | 14.0 | 13.0 | 13.8 |
| 36 | 13.5 | 13.7 | 13.2 | 13.6 |
| 37 | 12.8 | 14.0 | 13.1 | 14.2 |
| 38 | 13.8 | 14.4 | 13.5 | 14.4 |
| 39 | 14.2 | 15.0 | 14.4 | 15.2 |
| 40 | 14.7 | 13.4 | 14.4 | 13.3 |
| 41 | 15.5 | 16.0 | 15.2 | 15.8 |
| 42 | 13.8 | 13.2 | 13.5 | 13.1 |
| 43 | 14.3 | 15.0 | 14.6 | 15.2 |
| 44 | 13.9 | 15.2 | 14.2 | 15.4 |
| 45 | 15.2 | 15.8 | 14.8 | 15.5 |
| 46 | 11.6 | 12.7 | 11.4 | 12.4 |
| 47 | 12.3 | 14.0 | 12.5 | 14.3 |
| 48 | 12.5 | 13.4 | 12.6 | 13.7 |
| 49 | 13.2 | 13.5 | 13.2 | 13.6 |
| 50 | 14.6 | 15.6 | 14.4 | 15.6 |
| 51 | 12.8 | 13.6 | 12.6 | 13.4 |
| 52 | 14.2 | 14.8 | 14.4 | 14.8 |
| 53 | 13.5 | 13.7 | 13.3 | 13.9 |
| 54 | 12.4 | 14.2 | 12.4 | 13.8 |
| 55 | 13.2 | 14.5 | 13.4 | 14.3 |
| 56 | 13.3 | 13.8 | 13.4 | 13.6 |
| 57 | 11.5 | 12.5 | 11.6 | 12.8 |
| 58 | 12.8 | 14.2 | 13.0 | 14.1 |
| 59 | 13.5 | 13.9 | 13.4 | 13.8 |
| 60 | 14.2 | 15.6 | 14.2 | 15.5 |
